# Kyphoplasty of Osteoporotic Fractured Vertebrae: A Finite Element Analysis about Two Types of Cement

**DOI:** 10.1155/2019/9232813

**Published:** 2019-04-22

**Authors:** Carolin Meyer, Kerstin van Gaalen, Tim Leschinger, Max J. Scheyerer, Wolfram F. Neiss, Manfred Staat, Lars P. Müller, Kilian Wegmann

**Affiliations:** ^1^University Hospital of Cologne, Department of Orthopedics and Trauma Surgery, Kerpener Straße 62, 50937 Cologne, Germany; ^2^College of Biomedical Engineering, RWTH Aachen, Templergraben 55, 52056 Aachen, Germany; ^3^University of Cologne, Institute of Anatomy, Joseph-Stelzmann Str. 9, 50931 Cologne, Germany; ^4^University of Applied Science, FH Aachen, Heinrich-Mußmann Str. 1, 52428 Jülich, Germany

## Abstract

If conservative treatment of osteoporotic vertebral compression fractures fails, vertebro- or kyphoplasty is indicated. Usually, polymethylmethacrylate cement (PMMA) is applied coming along with many disadvantageous features. Aluminum-free glass-polyalkenoate cement (GPC) appears to be a benefit alternative material. This study aimed at comparing the mean stress values in human vertebrae after kyphoplasty with PMMA and GPC (IlluminOss™) at hand of a finite element analysis. Three models were created performing kyphoplasty using PMMA or IlluminOss™, respectively, at two native, human lumbar vertebrae (L4) while one remains intact. Finite element analysis was performed using CT-scans of every vertebra. Moreover the PMMA-treated vertebra was used as a model as analyses were executed using material data of PMMA and of GPC. The unimpaired, spongious bone showed potentials of 0.25 MPa maximally. After augmentation stress levels showed fivefold increase, rising from externally to internally, revealing stress peaks at the ventral border of the spinal canal. At central areas of cement 1 MPa is measured in both types of cement. Around these central areas the von Mises stress decreased about 25-50% (0.5-0.75 MPa). If workload of 500 N was applied, the stress appeared to be more centralized at the IlluminOss™-model, similar to the unimpaired. Considering the endplates the GPC model also closely resembles the unimpaired. Comparing the PMMA-treated vertebral body and the GPC-simulation, there is an obvious difference. While the PMMA-treated model showed a central stress peak of 5 MPa, the GPC-simulation of the same vertebral body presents lower stress of 1.2-2.5 MPa. Finite element analysis showed that IlluminOss™ (GPC), used in kyphoplasty of vertebral bodies, creates lower level stress and strain compared to standardly used PMMA, leading to lower stress concentrations on the cranial and caudal vertebral surface especially. GPC appears to own advantageous biological and clinical relevant features.

## 1. Introduction

Osteoporosis related fractures of the spinal column lead to pain and functional limitations, or even resulting in confinement to bed [1]. Due to the commonly wedge-shaped collapsing pattern of osteoporotic vertebral compression fractures (VCF) imbalanced kyphosis may occur, leading to loss of sagittal balance, chronic pain, and potentially resulting in decreased lung capacity and gastro-intestinal dysfunction [2, 3]. Controversial discussion about the appropriate individual treatment of VCF persists [4]. According to the treatment guidelines of Anselmetti et al. surgical treatment should be performed, if conservative treatment fails [5]. If the vertebral body is already deformed, kyphoplasty aims at correction of the vertebral shape in order to reduce pain and disability and to restore sagittal balance [1, 4–7].

It is commonly known that, besides shape of the fractures, the elastic modulus of the fracture region and of the implemented bone cement are important variables influencing short- and long-term outcome after kyphoplasty [8].

Commonly, polymethylmethacrylate cement (PMMA) is applied to the vertebra at kyphoplasty, in order to support vertebral structure. PMMA however comes along with specific disadvantageous characteristics. PMMA generates significant heat during the exothermic hardening process and it lacks biologic integration and healing due to induction of chemical or thermal necrosis within the vertebral body [9]. Moreover, the elastic modulus of PMMA exceeds the one of cancellous bone up to the 30-fold [1, 9–11]. This mismatch of mechanical properties might contribute to significantly higher onset of vertebral fractures adjacent to augmented levels [4, 12]. The mathematical relation between the elastic modules of bone and cement correlates directly proportional to stress and strain appearing during loading of augmented vertebrae [13]. Therefore, an alternative material for augmentation of VCF with an elastic modulus conforming to that of native human bone could be of interest.

In dental medicine aluminum-free glass-polyalkenoate cement (GPC) showing an elastic modulus closer to native bone has been used for stabilization and fixation of dental implants for decades [14–16]. On the basis of GPC the IlluminOss™ photodynamic Bone Stabilization System™ (IlluminOss™ Medical Inc., 993 Waterman Avenue, East Providence, RI 02914, USA) was developed and has already been in clinical use for fixation of long bones of the upper and the lower extremity and at the pelvis [14–16].

In order to examine if GPC offers advantages concerning stress and strain during loading of augmented vertebrae, the present study aimed for comparing mean stress values in the endplate of human cadaveric vertebral bodies after kyphoplasty with PMMA and GPC (IlluminOss™) performing finite element analyses.

## 2. Materials and Methods

Three fresh frozen, human lumbar vertebrae (L4) were available for investigation. IRB approval compliant to the declaration of Helsinki was obtained from our institutional ethical review committee.

Specimens were taken from two female and one male donors with an average age of 80 years (range 71–88). The specimens were excised from the lumbar spine after soft tissue release. The vertebrae were checked for osteolytic lesions or traumatic changes on X-ray amplification.

One specimen was left native as control specimen (V1), whereas two vertebrae were augmented performing kyphoplasty. In the second vertebra PMMA was used for augmentation (V2 PMMA), whereas kyphoplasty was performed using GPC, or rather IlluminOss™, in the third vertebra (V3 GPC). Preparing V2 PMMA, transpedicular drilling was performed using a 3.2 mm drill into the center of the vertebral body. The position of the drill was verified by fluoroscopy. Subsequently, trocars were placed and balloons were inserted into the vertebral body of V2 bipedicularly. Fluoroscopy was used to verify that the tip of the balloon reached the anterior aspect of the vertebral body, proofing correct positioning of the balloons. Afterwards, the balloons were inflated fluoroscopy-guided using 2 ml of contrast medium, respectively. Contrast medium and balloons were discharged and removed. Augmentation was performed successionally as PMMA-cement (Vertecem, Synthes Inc., Oberdorf, Switzerland) was standardly introduced into the vertebral body (V2 PMMA). During spontaneously ongoing cementing procedure of PMMA fluoroscopy was used to confirm the correct distribution of the cement within the cancellous bone. Preparing V3 GPC, transpedicular drilling was performed in the same way using a 3.2 mm drill into the center of the vertebral body. The position of the drill again was verified by fluoroscopy. Subsequently, Dacron balloons were inserted into the vertebral body of V3 bipedicularly. Fluoroscopy was used to verify that the tip of the balloon reached the anterior aspect of the vertebral body. Balloons were inflated fluoroscopy-guided using the biocompatible photodynamic liquid monomer (IlluminOss™), respectively. Polymerisation process of GPC was started by activating a light source connected to the Dacron balloons, working at a wavelength of 436 nm, after cement distribution was found correct by the investigators [14]. According to the literature a cement volume of 30 % relative to the volume of the vertebral body, meaning 4-6 ml for each vertebral body, was infused by manual pressure in V2 PMMA and V3 GPC [10, 19].

After finalization of augmentation, computed tomography (CT) scans of V1-3 were achieved and dicom-data were used to create finite element models of V1, V2 PMMA, and V3 GPC. In order to build a finite element model, CT data were applied to preprocessing software. Combining CT data and characteristic values of the elastic moduli and the Poisson ratio of cortical and cancellous bone as well as of cement types, a network of finite elements and junctions was generated, when a crosslinking algorithm was supplied (Table 1) [17, 18]. Thus, a three-dimensional model consisting of tetra-, penta-, and hexahedrons was created. After the three models were conducted to the finite element software, stress and strain distributions were generated using a numeric solver. Finite element models V1, V2 PMMA, and V3 GPC were analyzed with a workload of 500 N applied. The induced stress was measured in von Mises stress (MPa), which was calculated using the following equation:

(1) $$\begin{array}{c} \sigma_{v}=\sqrt{\frac{1}{2}\left[{\left(\sigma_{1}-\sigma_{2}\right)}^{2}+{\left(\sigma_{2}-\sigma_{3}\right)}^{2}+{\left(\sigma_{3}-\sigma_{1}\right)}^{2}\right]} \end{array}$$

Von Mises effective stress is commonly used as a predictive value for failure of isotropic ductile materials as to describe the stress situation in bone [1, 8, 10, 20].

**Figure 1:**
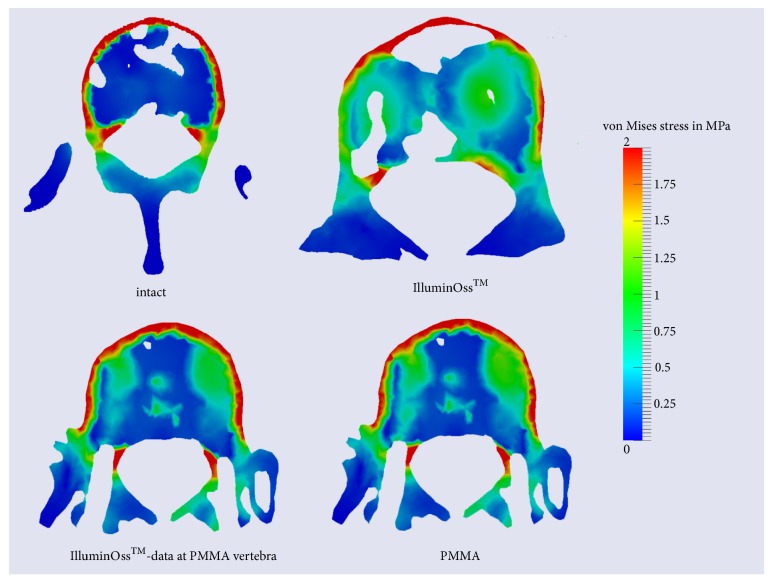
Stress distribution assessed at the intact vertebra, at the cemented vertebrae, and at one cemented vertebra analyzed using the material data of PMMA and GPC,* transversal cross sections*.

**Figure 2:**
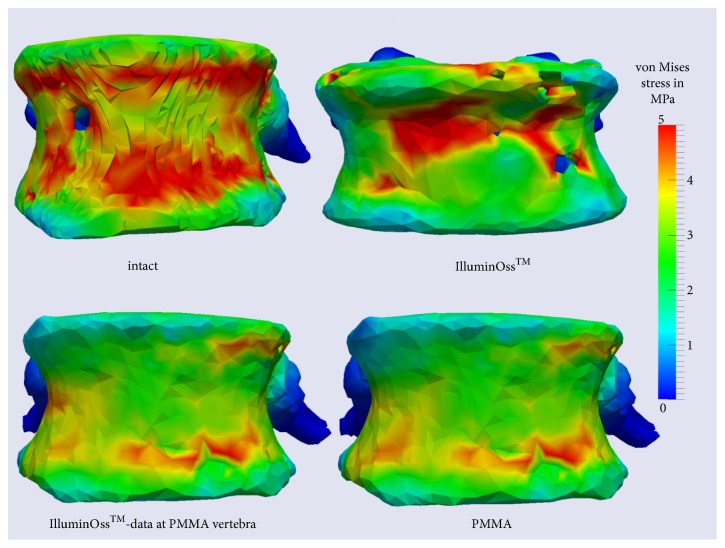
Stress distribution assessed at the intact vertebra, at the cemented vertebrae and at one cemented vertebra analyzed using the material data of PMMA and GPC,* coronal cross sections*.

**Figure 3:**
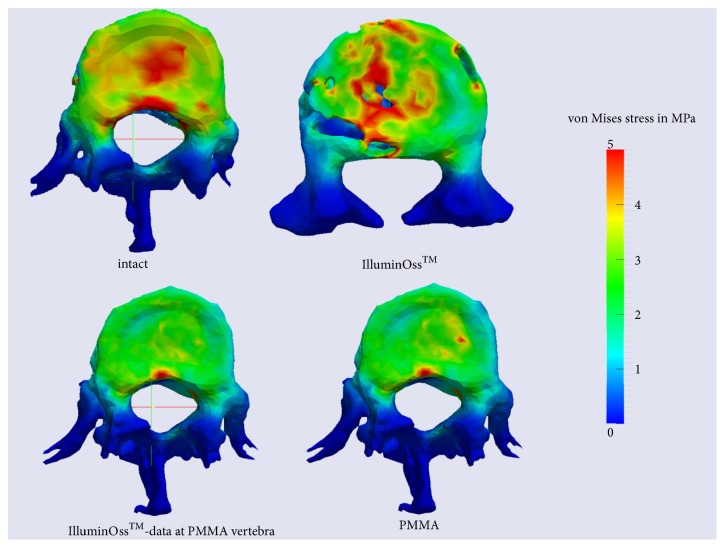
Stress distribution assessed at the intact vertebra, at the cemented vertebrae and at one cemented vertebra analyzed using the material data of PMMA and GPC,* transversal cross sections of the baseplates*.

Afterwards postprocessing was performed, in order to visualize the deformed model as well as stress and strain arising during deformation process using clear color scales [13, 21]. Evaluation and interpretation of generated data (V1-3) were performed on the basis of those color scales (Figures 1–3).

For creation of finite element models and analyses AMIRA 3D software vers. 5.4, FEI Company, Oregon/USA, was used for segmentation of CT data, and finite element analysis was done by “Code-Aster” (Électricité de France). Preprocessing and postprocessing were performed with SALOME versions 6.4 and 7.5 (Open Cascade, Gyancourt, France; Électricité de France; CEA).

In order to advance the comparability of the cements by decreasing possible bias due to regional morphological variations of the vertebrae and possible minor differences in the cement distribution, further analysis was performed using only finite element model V2 PMMA thereafter. As the skeleton of V2, which was augmented with PMMA in the first step, had already been analyzed using material data of PMMA, analysis was performed, again applying material data of GPC (V2 GPC) (Table 1). Thus, different shape, bone thickness, and cement distribution of the vertebral bodies were not integrated into the analysis.

## 3. Results and Discussion

Figures 1–3 demonstrate the analyzed stress within the different areas of the vertebral body and the areas of maximal strain of the different cement types.

Within the cancellous bone of the uncemented vertebral body, V1, the stress assessed increased up to 0.25 MPa maximally (Figure 1). Coronal cross sections as well as transversal cross sections of V1 revealed that stress amounted to at least 5 MPa at the cortical bone of the ventral border and of the dorsal border of the vertebral body, when workload of 500 N was applied (Figures 1 and 2). At the endplates, V1 presented stress of 1.2 up to 5 MPa (Figure 3).

### 3.1. Changes of Stress Distribution within the Cancellous Bone

After augmentation stress levels at the cancellous bone of the cemented vertebral bodies V2 and V3 showed a fivefold increase. As represented in transversal cross sections (Figure 1) only little difference regarding the arising stress was detected between V2 PMMA and V3. At the central areas of cement 1 MPa was measured in both, but V2 PMMA showed a peak of 1.4 MPa at the PMMA zone. Nevertheless, central area of maximal stress took a smaller place at V3. Around these central areas, there were small seams, where the von Mises stress decreased about 25-50 % to 0.5-0.75 MPa.

### 3.2. Changes of Stress Distribution within the Cortical Bone

All finite element models (V1-3) revealed stress peaks up to 5 MPa at the ventral border of the spinal canal, respectively at the dorsal border of the vertebral body, as well as up to >5 MPa at regions of cortical bone at the ventral border of the vertebrae. Nevertheless, at the ventral border of the vertebral model V2 PMMA, there appeared a decrease of stress to 2,5 MPa in the central area. Analyses of V3 showed similarity with the native V1. Presenting pressure peaks of 5 MPa comparably to the native model, however, the stress appeared to be more centralized at the GPC-treated model V3 (Figure 2).

### 3.3. Changes of Stress Distribution at the Endplates

Analyzing the endplates, V3 also closely resembled the native V1. The endplate of V3 featured stress values from 2.5 up to 5 MPa. In V2 and V3 the stress rose from externally to internally (Figure 3), but in V2 PMMA stress was minimized overall.

### 3.4. Comparison of V2 PMMA and V2 GPC

Axial cross sections show that the central green, representing the area of maximal stress, was smaller at V2 GPC as at V2 PMMA (Figure 1). Regarding the cortical borders of the vertebra, there appeared a decrease of stress to 2,5 MPa in the central area of the ventral border of the vertebra despite different cement types in V2 PMMA and V2 GPC (Figure 2). Regarding stress levels at the endplates, stress was minimized overall in V2 PMMA as well as at V2 GPC. While V2 PMMA showed less stress but a peak of 5 MPa, V2 GPC presented lower stress values of 1.2 to 2.5 MPa (Figure 3).

The present finite element analysis, on stress distribution within the vertebral body after kyphoplasty demonstrates stress values clearly differing in cement augmented vertebral bodies from native ones. Maximal stress peaks, similar to values assessed at cortical regions, were found at the center of PMMA and GPC cements. Areas of maximum stress values within cancellous bone were smaller in GPC models. The stress decreased 25-50 % at the outer cemented area within the GPC models in contrast to PMMA models. Analyses of the stress at the cortical bone did not show any significant difference. Moreover GPC caused stress levels half as low again than PMMA at the endplates.

Rohlmann et al. documented a maximum of 1,5 MPa in cancellous bone and of 31,1 MPa in cortical bone simulating a load of 500N at the treated vertebrae [8]. Regarding the stress within the cancellous bone, values of the current investigation match the ones reported by Rohlmann et al. However, the maximum stress of 5 MPa, currently measured at the cortical bone, was sixfold less than seen by the colleagues [8].

Previous finite element analyses of intact and augmented vertebrae revealed that the von Mises stress within the cortical bone decreases about 50 % due to augmentation. These measurements corroborate the belief that cement applied to a vertebra acts as a buttress. The internal stress at the cancellous bone increases, resulting in a decrease of stress at the cortical bone [10, 22]. As stress values within the cancellous bone appeared a little smaller in the GPC model than in the PMMA one, the “buttress-effect” might be reduced by the use of GPC.

In 2005 Rohlmann et al. analyzed the effect of bone cement application into intact as well as into fractured vertebrae [1]. It was displayed that baseplates' plastic deformity decreases with an increase of stiffness of the cancellous bone, as induced by augmentation, resulting in an increase of stress and prevalent pressure at the intervertebral disc [1]. Baroud et al. assessed that baseplates' deformity of augmented vertebrae is reduced to 7 % and the stiffness affecting the baseplate increases about 17 % in contrast to native vertebrae. Authors pointed out that the intradiscal pressure rises about 19 %, consecutively, adjacent to augmented vertebrae [10]. Similar results were published by Polikeit et al. 2003. Using finite element method, authors figured out that the von Mises stress raises about 5 % at the surrounding cancellous and cortical bone and that the stiffness affecting the baseplate increases about 13 %, if a vertebral body is augmented and compression forces are applied [22]. In the current investigation attention was not focused on the effects to the intervertebral disc. However, as a consequence of the current results it seems probable that the intradiscal stress might be reduced using GPC instead of PMMA.

The occurrence of adjacent level fractures in patients suffering from osteoporosis remains an issue of controversial discussion. Previous investigations figured out that the relative risk of adjacent level fractures after vertebroplasty is increased nearly fivefold compared to that for nonadjacent [11, 12, 23]. Many authors support the opinion that cement used for kyphoplasty acts as an internal fixation but that the risk of adjacent level fractures has to be decreased due to reduction of the flexion moment to the surrounding vertebral bodies after restoration of the anterior column performing kyphoplasty [24–26]. Previous biomechanical investigations showed that vertebral augmentation with a clinically relevant volume of cement does not result in stress peaks under the endplate [1, 27]. Present results conform to these findings.

Demonstrating missing stress peaks at the endplates, Aquarius et al. concluded that the higher amount of adjacent level fractures observed is not caused by the cement itself [27]. Rohlmann et al. agreed stating that fracture's shape, cement volume applied, and elastic modulus of the fracture region and of the implanted bone cement are most important variables influencing short- and long-term outcome after kypho- or vertebroplasty [8]. Fracture's shape and elastic modulus of the fracture region always remain an individual variable. Regarding cement volume applied, it is commonly known that it has to count 30 % relative to the volume of the vertebral body, meaning 4-6 ml for each vertebral body [10, 19]. Concerning cement distribution, Chevalier et al. pointed out that cement fillings touching both endplates have to be avoided resulting in an up to eightfold increased stiffness as to twelvefold increased strain [28].

As it is commonly known that elastic moduli of human bone and PMMA differ clearly, the question about a kind of bone cement, whose elastic modulus resembles the native bone one's, still remains. Few investigations were performed searching for such kind of cement: Schulte et al. compared biomechanical effects of PMMA and silicone cement used for kyphoplasty. They showed that silicone cement has the biomechanical potential to reduce secondary fractures [20]. Dickey et al., testing different kinds of cement, concluded that, in comparison to conventional augmentation materials, the use of aluminum-free GPC acts similar to healthy bone and could decrease the risk of adjacent fractures [27].

Overall, in a synopsis of the literature and present results we conclude that GPC creates lower level of stress and therefore leads to lower strain compared to standardly used PMMA, if workload is applied to the cemented vertebra. Accordingly, plastic deformity of the endplates might be reduced less and stress levels at surrounding intervertebral disc might be reduced more than in the use of PMMA. Moreover, elastic modulus of GPC resembles the native bone one's more than the elastic modulus of PMMA, which may be advantageous with respect to biomechanical changes and strain to the adjacent spinal levels as Dickey et al. supposed [27].

Besides, there are further advantages in the use of GPC instead of PMMA. As the risk of leakage is reduced due to the fact that the cement is inserted into the placed balloon, the hardening process of this type of cement is completely under the surgeon's control. Polymerisation of the monomer only proceeds when light with a wavelength of 436 nm is simultaneously present. The reconstruction of height of the vertebral body as well as of the endplates can be controlled better as the surgeon is able to define the point in time to execute the hardening process [9, 14]. Interdigitation of GPC and the surrounding bone, however, is reduced, because of the confined balloon space. Moreover, PMMA shows a strong exothermic reaction (up to 40°C – 100°C), possibly burning on tissues near the vertebral body or vertebral bone cells themself. In cancer induced fractures this exothermic reaction happens to be beneficial. On the contrary, in benign, osteoporotic fractures the heat is rather disruptive, as it potentially disturbs the naturally started healing process of a fracture [29]. At the hardening process of GPC there is no relevant exothermic reaction (maximum 40°C), which may damage the surrounding tissue [9, 14].

We acknowledge several limitations of the current investigation. Fresh frozen vertebrae were augmented in vitro and fresh frozen vertebral bodies have been used. The matter how far bone stiffness of fresh frozen bones is different to natural ones has not been clarified and remains contentious in literature [30]. Furthermore stress (Pa) at the cemented vertebrae was only evaluated in an in vitro setup and material properties were simplified. In vivo there is, of course, an important influence of ligaments and muscles as well as of the intervertebral disk to the load pressure distribution. These biomechanically considerable structures were not taken into account in this investigation. Additionally, it has to be mentioned that previous measurements of bone density are missing. Therefore the comparability of the vertebrae remains unclear. Against this background, further finite element model was built using the PMMA-treated vertebra and the material data of GPC in order to achieve comparability (V2 PMMA and V2 GPC).

Further biomechanical testing of motion segments of the spine including a vertebra cemented with GPC also with regard to the surrounding tissue is desirable. Moreover, a definitive statement about the aspect of stabilization cannot be given as a result of this investigation, as stability and fatigue behavior of the different types of augments were not analyzed. Further investigation has to be performed, in order to analyze if GPC provides appropriate stability to the cemented vertebra. Those investigations are essential, in order to get to know, if GPC really represents an alternative material in augmentation of VCF. Afterwards clinical research is necessary to assess osseous growth and the cement's integration within the vertebrae.

## 4. Conclusions

GPC creates lower level stiffness and lower stress within the cancellous bone compared to standardly used PMMA. Even stress levels at the cortical bone of a vertebra cemented with GPC resemble stress values of native vertebra more than after augmentation with PMMA. Further biomechanical and clinical testing is necessary to prove the stability provided by the cement and the effects on the motion segments below at the spine.

## Figures and Tables

**Table 1:** Material data used for finite element analysis [17, 18].

	E-modulus E [MPa]	Poisson's ratio *ν*
Cortical bone	10000	0,3
Cancellous bone	100	0,25
PMMA	2700	0,3
GPC	1200	0,3

## Data Availability

The data used and analyzed during the current study are available from the corresponding author on reasonable request.
